# A Chloroplast-Localised Fluorescent Protein Enhances the Photosynthetic Action Spectrum in Green Algae

**DOI:** 10.3390/microorganisms10091770

**Published:** 2022-09-01

**Authors:** Julio V. Suarez, Elisabeth A. Mudd, Anil Day

**Affiliations:** 1 School of Biological Sciences, University of Manchester, Michael Smith Building, Oxford Road, Manchester M13 9PT, UK; 2 Facultad de Ciencias, Universidad Autónoma de Baja California, Carr. Transpeninsular 3917, Ensenada 22860, Mexico

**Keywords:** chloroplast transformation, expanding photosynthetic action spectrum, green microalga, Katushka fluorescent protein, photosynthetically active radiation, PAR

## Abstract

Green microalgae are important sources of natural products and are attractive cell factories for manufacturing high-value products such as recombinant proteins. Increasing scales of production must address the bottleneck of providing sufficient light energy for photosynthesis. Enhancing the photosynthetic action spectrum of green algae to improve the utilisation of yellow light would provide additional light energy for photosynthesis. Here, we evaluated the Katushka fluorescent protein, which converts yellow photons to red photons, to drive photosynthesis and growth when expressed in *Chlamydomonas reinhardtii* chloroplasts. Transplastomic algae expressing a codon-optimised Katushka gene accumulated the active Katushka protein, which was detected by excitation with yellow light. Removal of chlorophyll from cells, which captures red photons, led to increased Katushka fluorescence. In yellow light, emission of red photons by fluorescent Katushka increased oxygen evolution and photosynthetic growth. Utilisation of yellow photons increased photosynthetic growth of transplastomic cells expressing Katushka in light deficient in red photons. These results showed that Katushka was a simple and effective yellow light-capturing device that enhanced the photosynthetic action spectrum of *C. reinhardtii.*

## 1. Introduction

Microalgae have evolved over billions of years into a successful and widespread group of photosynthetic eukaryotes [1]. They are sources of natural products, including carotenoids and long-chain polyunsaturated acids, which are used as food additives and nutraceuticals [2,3]. Microalgae have attracted considerable interest as sources of biofuels [4,5,6] and sustainable lean-carbon production platforms for expressing vaccines and biopharmaceutical proteins [7,8]. Maximising biomass is key to the production of all high-value products expressed in microalgae [9]. At high cell densities and in deep water, biomass accumulation is limited by light availability [10]. Synthetic biology provides a route towards enhancing light-capture systems in microalgae to boost growth in limiting light [9]. *Chlamydomonas reinhardtii* provides a suitable algal model for synthetic biology projects by allowing the design of algal cells with new functionalities, for example nitrogen fixation, and pharmaceutical protein and vaccine production [7,8,11,12,13].

Light penetration into dense algal cultures can be improved by reducing light-harvesting antennae sizes, which decreases chlorophyll content [14,15,16]. This improves algal biomass production at high light intensities but is a disadvantage in low light due to impaired light harvesting by short antennae [17]. In green algae and plants, photosynthetically active radiation extends from 400 nm to 700 nm. This provides opportunities to expand the photosynthetic action spectrum by developing new light-harvesting mechanisms to capture photons with wavelengths below 400 nm (UV) and above 700 nm (far red) [16,18,19]. Sunlight at the Earth’s surface contains more far-red photons than UV photons [19,20], providing an advantage for engineering far-red photon (701 nm to 750 nm) collection systems in terrestrial plants [15,20,21,22]. Opportunities to enhance the photosynthetic action spectrum are not limited to far-red photons, which have been the focus of a number of studies [21,22]. Yellow photons (~570 nm to ~590 nm) are abundant in the photon flux spectrum of solar radiation at the Earth’s surface [20] but correspond to a trough that lies between ~550 nm to ~600 nm in the absorption spectrum of green algae [23]. Importantly, yellow photons penetrate water to greater depths than far-red photons [15,24]. This makes the capture of yellow photons more attractive than harvesting far-red photons for expanding the photosynthetic action spectrum in aquatic green algae.

Here, we designed a yellow photon-capturing device based on a fluorescent protein to drive photosynthetic growth. The device was built and tested in *C. reinhardtii*. It was based on the observation that corals express fluorescent proteins capable of converting blue and green photons, which predominate in deep water, to longer wavelength photons which are scarce at these depths [25,26]. This light conversion to longer wavelengths may have a role in increasing photosynthesis in the symbiotic zooxanthellae present in corals [25,26], although other functions for fluorescence have been proposed [27,28].

**Figure 1:**
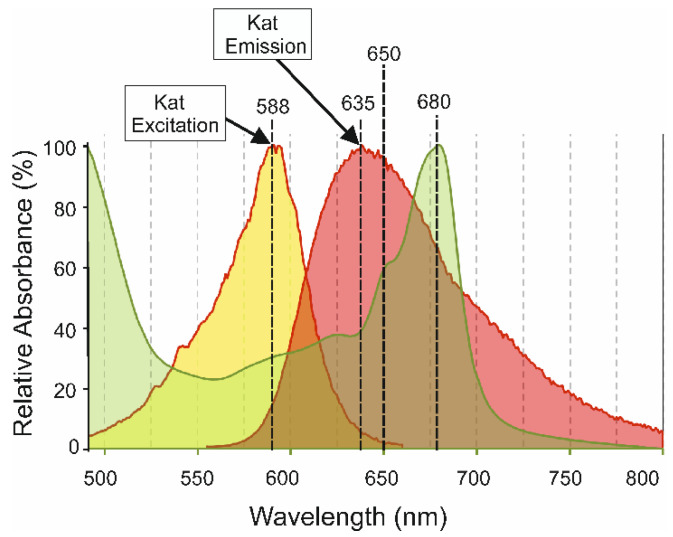
Scheme showing the locations of the excitation (588 nm) and emission (635 nm) peaks of the red fluorescent Katushka (Kat) protein [30] relative to the absorption spectrum (green) of *C. reinhardtii* cells (not to scale). The absorption spectrum of cells was normalised to the 680 nm peak.

We searched the palette of fluorescent proteins [29] for a suitable candidate capable of converting yellow photons to red photons. The far-red fluorescent protein Katushka, also known as TurboFP 635, is a 231 amino acid, 26 kDa protein that absorbs yellow photons with an excitation peak at 588 nm and emits red light with an emission peak at 635 nm [30]. These 588 nm excitation and 635 nm emission peaks overlap with the ~550 nm to ~ 600 nm trough and red peaks in the absorption spectrum of *C. reinhardtii* cells [23], respectively (illustrated by Figure 1). The high quantum yield of 0.34, brightness of 22.1, photostability and pH tolerance of Katushka [30] make it preferable to similar fluorescent proteins such as mPlum with 590 nm excitation and 649 nm emission peaks [31]. Crucially, Katushka fluorescence exhibits an eight-fold increase in brightness between 650 nm to 800 nm compared to mPlum [30], thereby providing more energy for chlorophyll absorption and photosynthesis. Expression in chloroplasts rather than the cytosol ensured that Katushka was located in relatively close proximity to the light-harvesting complexes in the thylakoid membranes of chloroplasts. This increases the efficiency of capture of red photons by chlorophyll. The use of Katushka as a light-capturing device for enhancing photosynthesis was validated by the observation that transplastomic algae expressing Katushka showed improved photosynthetic growth in yellow light compared to control strains.

## 2. Materials and Methods

### 2.1. Chlamydomonas Strains and Media

Chlamydomonas strains CC-373 (ac-u-c-2-21, *atpB* deletion, mt+) and CC-503 (cw92 mt+) were from the Chlamydomonas Resource Center (University of Minnesota; https://www.chlamycollection.org/ (accessed on 18 July 2022)). Non-photosynthetic strain CC373 [32,33] was maintained on Tris-Acetate-Phosphate (TAP) media [34]. Strains CC-503 and transplastomic strains were maintained on minimal media lacking acetate [34]. Photosynthetic strains were maintained at 20 °C in 30 μMoles m^−2^ s^−1^ light (cool white L36W/640 lamps, Osram, St Helens, UK) in a 12 h light/dark cycle.

### 2.2. Nucleic Acid Manipulations

Standard molecular biology and recombinant cloning procedures were followed [35]. Unless otherwise indicated, all PCR reactions were carried out in a 20 μL final volume using 0.6 U of Vent DNA polymerase (New England Biolabs, Hitchin, UK), 200 μM final concentration of deoxynucleotides (Bioline, London, UK), and 500 nM final concentration of primers (Sigma-Aldrich, Poole, UK). Primer sequences are listed in Supplementary Table S1. Restriction enzyme digests used 1 μg of DNA under manufacturer’s conditions (New England Biolabs, Hitchin, UK, or Roche, Welwyn Garden City, UK) except for *Kpn* I partial digestions, where 5 μg of DNA was used. Total DNA from Chlamydomonas was prepared according to Day et al. (1988) [36]. Sequences were visualised in silico using Vector NTI Advance v 11.5 (Invitrogen, Carlsbad, CA, USA) and Geneious Prime (Biomatters, Auckland, New Zealand). The wild-type (Acc no. BK0054) and CC-373 (MF083692) chloroplast genomes were aligned using MAFFT [37] in Geneious Prime using the default parameters to locate the deletions.

### 2.3. Synthesis of the CpKat Gene

The 231 amino acid Katushka fluorescent protein was back-translated in silico using vector NTI (Invitrogen, Carlsbad, CA, USA) with frequent codons in *C. reinhardtii* chloroplast genes [38]. A glycine GGT codon was inserted after the N-terminal methionine to create an *Nco I* site (Supplementary Figure S1). The resulting coding sequence, named cpKAT, was assembled by stepwise overlap PCR using thirty-two oligonucleotides of 34 to 38 nucleotides in length (oligonucleotides 783 to 815). Ten complementary nucleotides allowed assembly of dimers; 17 base overlaps between adjacent dimers enabled synthesis of 15 tetramers, which were mixed to assemble the complete gene. For dimers, reaction mixes were prepared to contain 90 mM final concentration of each of the two oligonucleotides and 1.25 mM final concentration of each deoxyribonucleotide in restriction enzyme buffer A (Roche Life Sciences, Burgess Hill, UK). Reaction mixes were denatured for two minutes at 96 °C and then cooled down to 37 °C. Next, 0.37 U of Klenow enzyme (Roche, Lewes, UK) was added to each mixture and incubated for 1 h at 37 °C and 16 h at 15 °C. Fifteen 100 to 103 bp long tetramers were then assembled from adjacent dimers by eight cycles of PCR using a 34 °C annealing step and Vent DNA polymerase (New England Biolabs). Finally, the full-length CpKat gene was assembled by 20 cycles of PCR amplification with BiomixRed master mix (Bioline) by combining all fifteen 100 to 103 bp long tetramers and increasing the concentration of external primers 782 and 815. The complete 699 bp CpKAT gene with TAA stop codon was purified by agarose gel electrophoreses and cloned into *E. coli* vector pGEM-T easy (Promega, Southampton, UK).

### 2.4. Construction of the pB10 Chloroplast Transformation Vector

*C. reinhardtii* chloroplast Bam10 fragment (7.6 kb) in pBR313 [39] from the Chlamydomonas Resource Center was used as a template to amplify the 2.5 kbp right targeting arm with primers 862 and 863, which was then digested with *Xba I* to *Kpn I* and cloned into pBluescript [40] to produce pBluescript-Bam10-Right. For the left targeting arm, a 3.5 kbp *Bam HI* to *Pst I* fragment of Bam10 was cloned into pUC18 [41]. The cloned left arm was digested with *Bam HI* and partially digested with *Kpn I* to obtain a 2.1 kbp fragment which was subcloned into pBluescript using linkers comprising annealed oligonucleotides 1123 and 1124, and annealed oligonucleotides 1125 and 1126. The left arm was excised with *Sac I* and *Xba I* and inserted into pBluescript-Bam10-Right to produce chloroplast transformation vector pB10 containing both targeting arms separated by a polylinker with unique *Not I*, *Xba I*, *Apa I,* and *EcoRI* sites for inserting foreign genes. A 297 bp *rbcL* 3′UTR region amplified from pUC-atpX-*aadA* [42] with primers 864 and 865 was cloned into pGEM-T easy. pB10-AKL was produced by cloning CpKat as an *NcoI* to *PstI* fragment into pUC-atpX to produce atpX-CpKat. The *atpA* promoter and 5′UTR and CpKat were excised from atpX-CpKat using *EcoRI* to *PstI*, mixed with the ~300 bp *rbcL* 3′UTR excised with *PstI* and *NotI*, and ligated into *EcoRI-* and *Not I*-restricted pB10. The *psbD* promoter and 5′UTR was isolated by PCR from CC-503 total DNA using primers 5199a and 5199b, cloned into pUC18, then excised as a *HincII* to *NcoI* fragment to replace the *atpA* 5′ region excised with *Eco RI* and *Nco I* in pB10-AKL to form pB10-DKL. *Hinc II* and *Eco RI* ends were made blunt using Klenow enzyme and dNTPs.

### 2.5. Chloroplast Transformation

Chloroplast transformations were performed as described previously [43] using the Biolistic PDS-1000/He Particle Delivery System (Bio-Rad, Hemel Hempstead, UK) with 1 μM gold particles and conditions: 1100 psi rupture disks, 28 mm Hg vacuum, and 6 cm distance from the rupture disk to the lawn of cells on 9 cm Petri dishes containing minimal media. The pB10 constructs were transformed into strain CC-373 [43]. Bombarded plates were incubated at 25 °C with a light intensity of 200 μmol m^−2^ s^−1^ using cool white fluorescent lamps. Individual transformant colonies were picked after 2 weeks and serially propagated on minimal medium for six months.

### 2.6. PCR Analyses of Microalgae

DNA for PCR analyses was obtained as described by Cao et al. (2009) [44]. CpKat integration was verified by PCR using primers 784 and 1420. PCR products were size-fractionated by electrophoresis on tris-borate-EDTA 1% *w*/*v* agarose gels at 5 V cm^−1^.

### 2.7. Protein Blot Analyses

Procedures for extracting proteins from algal cells, SDS-PAGE, transfer of size-fractionated proteins to blots, and visualisation of specific antibody-bound bands are described in Suarez et al. (2014) [45]. The anti-tRFP rabbit polyclonal antibody specific for Katushka was from Cambridge Bioscience (Cambridge, UK).

### 2.8. Measurement of Dissolved Oxygen

Dissolved oxygen was calculated using the modified Winkler method [46] according to Labasque et al. (2004) [47], measuring absorbance at 466 nm in a Synergy HT multi-mode plate reader (Biotek Instruments, Winooski, VT, USA). Transplastomic cell cultures were adjusted to 1 × 10^6^ cells mL^−1^ density in minimal media during the dark-growth period in 100 mL shaking flasks. A total of 5 mL of culture were transferred to each of three 15 mL conical tubes and placed in 25 °C illuminated growth chambers with either 30 µmol m^−2^ s^−1^ cool white L36w/640 fluorescent lamps (Osram, Munich, Germany) or 30 µmol m^−2^ s^−1^ yellow (λ_max_ 590) LED lamps (Ecolight Ltd., Chichester, UK). Cells were left for 6 h and then cultures were fixed by adding 60 µL of 2% *w*/*v* sodium azide. A total of 2 mL of fixed cultures were transferred to 6-well microtiter plates for absorbance readings. Experiments were performed in triplicate. Total chlorophyll was measured by spectrophotometric measurements at 647 nm and 664 nm, according to Ritchie (2006) [48]. All experiments were carried out in the dark, and solvents and extracts were kept at 4 °C until measurements were carried out in a Synergy HT multi-mode plate reader.

### 2.9. Algal Growth Conditions

For each construct, three transplastomic lines from colonies derived from independent transformation events were used. Each line was cultured in triplicate providing nine cultures (three biological replicates × three technical replicates). Transplastomic cells were inoculated to an initial optical density of 0.1 at 750 nm (Jenway 7315 spectrophotometer, Fisher Scientific, Loughborough, UK) in 6 mL minimal media in 14 mL Falcon culture tubes (Fisher Scientific) and incubated at 25 °C in continuous light. Growth of the non-transgenic *C. reinhardtii* strain CC-503 was in 100 mL of minimal medium. Cells were grown for two weeks and 200 μL samples were taken for OD 750 nm readings using a Synergy HT microplate reader (Biotek UK, Potton). Lamps used were cool white L36W/640 fluorescent lamps (Osram); blue (λ_max_ 460), yellow (λ_max_ 590) and red (λ_max_ 660) LED lights (Ecolight Ltd., Chichester, UK). Light intensity received by each culture tube was measured using a SKP 200 photometer (Skye Instruments, Llandrindod Wells, UK).

### 2.10. Fluorescence and Spectral Analysis

Fluorescence measurements were performed in a Synergy HT microplate reader using 200 μL samples with a 590 nm/20 nm excitation filter and a 645 nm/40 nm emission filter. Cells growing in 200 μL of minimal media were bleached in 50 μM norflurazon (Sigma-Aldrich). Absorbance at 688 nm and 750 nm, and fluorescence (645 nm/40 nm) were measured hourly. All experiments were performed in triplicate. The absorption spectrum of *C. reinhardtii* CC-503 (Figure 1) was obtained from 1 mL of 1 × 10^7^ cells mL^−1^ grown in minimal medium using a Synergy HT microplate reader with a 1 nm bandpass filter and was similar to previous reports [23]. Absorption was normalised to the absorbance at 680 nm.

## 3. Results

### 3.1. Design of Chloroplast Transformation Vectors

**Figure 2:**
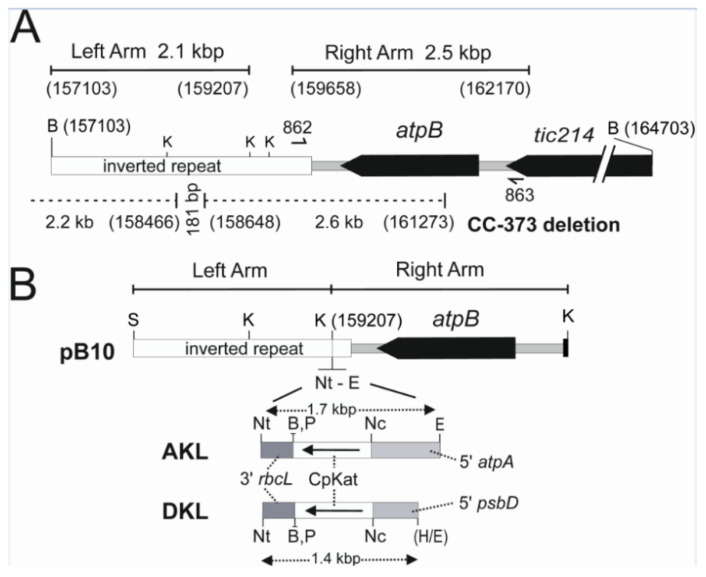
Chloroplast transformation vectors and integration site. (**A**) Map of *C. reinhardtii* Bam10 DNA fragment of *C. reinhardtii*. Shown are the targeting arms used to assemble vector pB10, primers 862 and 863 used to amplify the right targeting arm of pB10, region deleted (dotted line) in *atpB* mutant strain CC-373 [33], and restriction enzyme sites B—*Bam HI* and K—*Kpn I*. Chloroplast map co-ordinates (Acc no. BK000554) [38] are shown in brackets. (**B**) Map of *C. reinhardtii* chloroplast targeting vector pB10 showing Nt—*Not I* and E—*Eco RI* linker used to insert the AKL and DKL expression cassettes (Supplementary Figure S1) containing the CpKat coding region. The 5′ and 3′ regulatory regions are indicated. B—*Bam HI*, K—*Kpn I*, Nc—*Nco* I, P—*Pst I*, S—*Sac I*, H/E—*Hinc II*- *Eco RI* blunt ligation.

A synthetic Katushka coding region (CpKat) containing frequently used *C. reinhardtii* chloroplast codons was synthesised from oligonucleotides by overlap PCR. Transgenes were inserted downstream of the *atpB* gene in the large 4.5 kb intergenic region between the *atpB* gene in the single copy region and the *rrnS* gene in the large inverted repeat. This was carried out by cloning the 2.1 kb left and 2.5 kb right targeting arms from the 7.6 kb Bam 10 fragment (Figure 2A) and inserting transgenes into the *Kpn I* site located at co- ordinate 159207 of the chloroplast genome [38]. The AKL and DKL expression cassettes (Supplementary Figure S1) contained the CpKat coding region bordered by the promoter and 5′ UTR from the chloroplast *atpA* or *psbD* gene and a common 3′ regulatory region from the *rbcL* gene (Figure 2B). Chloroplast transformation vectors pB10-AKL and pB10-DKL were assembled by insertion of AKL and DKL expression cassettes into the linker sequence between the left and right targeting arms.

### 3.2. Isolation of CpKat Chloroplast Transformants Expressing Katushka

Selection of chloroplast transformants was based on repair of the deleted *atpB* gene in the non-photosynthetic CC-373 strain [33] with the wild-type *atpB* gene in pB10 [43]. This selection regime allowed the isolation of photosynthetic transformants on minimal media lacking acetate. Neither the vector nor the *C. reinhardtii* CC-373 strain used as a recipient for transformation contained antibiotic resistance genes. The absence of antibiotic resistance genes avoids the regulatory concerns associated with the presence of these genes in transgenic algae [49]. The chloroplast DNA deletion in CC-373 [33] extends into the inverted repeat (Figure 2A). Crossover events in the left 2.1 kbp and right 2.5 kbp targeting arms of the pB10 vector (Figure 2B) integrate the intact *atpB* gene and foreign genes into the CC-373 chloroplast genome. These crossover events take place in sequences common to the pB10 vector and CC-373 genome that flank the CC-373 deletion and correspond to a 181 bp sequence in the left arm and 897 bp sequence in the right arm. Although the CC-373 deletion extends beyond the left *Bam* HI site in Bam 10, the left junction of the deletion contains a 181 bp sequence containing multiple ACACTTTATTTT tandem repeats that is present in Bam 10 (location shown in Figure 2A). The empty pB10 vector and the two CpKAT vectors (pB10-AKL, pB10-DKL) were transformed into CC-373 by particle bombardment. Transplastomic lines from independent transformation events were selected from separated colonies growing photosynthetically on minimal media lacking acetate. Three AKL and DKL lines and two pB10 lines were selected for further analysis. These lines corresponded to the transplastomic algae transformed with the pB10-AKL, pB10-DKL, and empty pB10 vectors.

**Figure 3:**
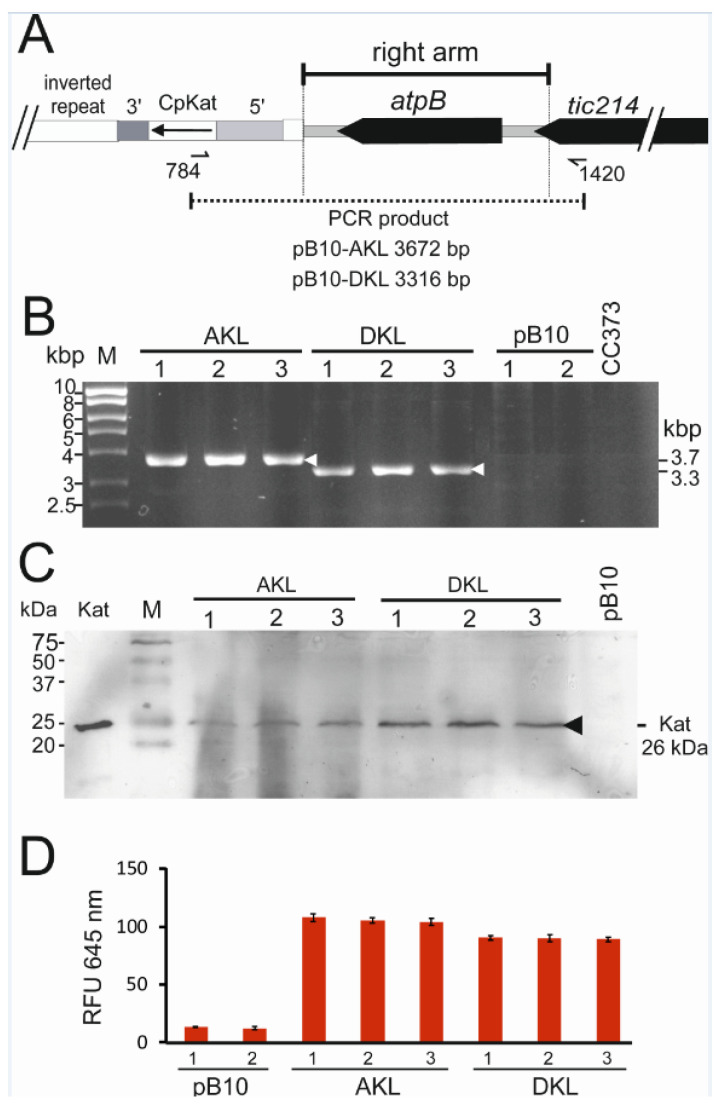
Verification of CpKat integration and expression in transplastomic algae. (**A**) Map of CpKat integrated into chloroplast DNA showing locations of inverted repeat, *atpB*, *tic214,* and PCR product sizes amplified by primers 784 and 1420. (**B**) PCR products amplified by primers 784 and 1420 from the indicated DNA samples fractionated by agarose gel electrophoresis. Sizes of PCR products and MW marker (M) bands are indicated. (**C**) Western blot analysis of CpKat transformant strains (AKL and DKL) and empty pB10 transformants (pB10) using a Katushka-specific antibody. Equal amounts of total cell protein were loaded per lane. *E. coli*-expressed Katushka (Kat) was used as a positive control. The sizes of MW standards (M) and Katushka are indicated. (**D**) Fluorescence (645 nm) of AKL, DKL, and pB10 cells excited with 590 nm light. Equal numbers of cells were used for each sample. Average from three replicate cultures with standard errors shown. RFU—relative fluorescence units.

All transgenic lines were propagated continuously on minimal medium for six months to select transgenic chloroplast genomes containing the intact *atpB* gene. Integration of the CpKat transgene in CC-373 transformants was confirmed by PCR analysis using primer 784 located in CpKat and primer 1420 located in chloroplast DNA beyond the right targeting arm. Primer locations are shown in Figure 3A. The predicted PCR products of 3.7 kbp and 3.3 kbp were amplified from pB10-AKL and pB10-DKL DNA, respectively (Figure 3B). The sizes of the PCR products resulted from the differing lengths of the 5′ *atpA* and *psbD* regulatory sequences. No PCR products were detected using DNA from pB10 transplastomic lines and CC-373, which lacked the CpKat transgene (Figure 3B).

Accumulation of the 26 kDa Katushka protein in AKL and DKL transplastomic lines was verified by protein blot analysis. A Katushka-specific antibody detected a single band of the correct size in total protein from AKL and DKL lines fractionated by denaturing SDS-PAGE (Figure 3C). The 26 kDa band co-migrated with recombinant Katushka overexpressed in *Escherichia coli* (Figure 3C, lane 1). No Katushka band was detected in the negative control (Figure 3C, last lane) corresponding to pB10 transplastomic algae lacking the CpKat gene.

Katushka fluorescence from cultures was detected by using an excitation wavelength of 590 nm and detecting fluorescence at 645 nm. Figure 3D showed that 645 nm fluorescence was significantly higher in the AKL and DKL lines compared to the pB10 line lacking the CpKat gene. This was consistent with correct folding and maturation of Katushka, which are required to form the active fluorescent tetramer [50].

### 3.3. Chlorophyll Absorption of Katushka Fluorescence

**Figure 4:**
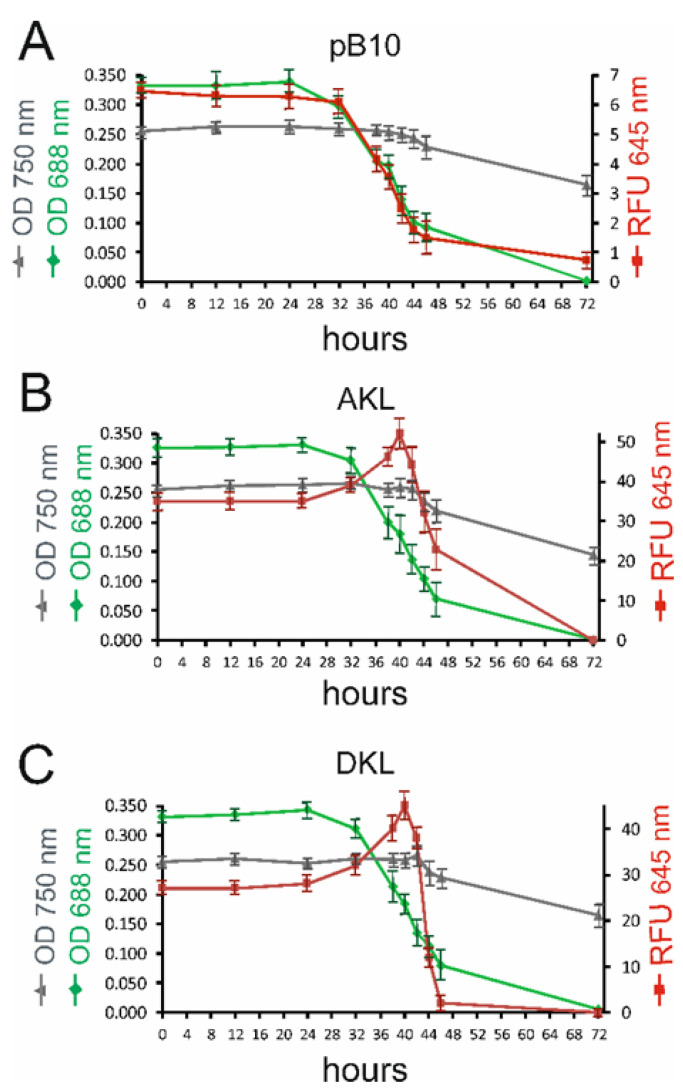
Monitoring cell density (750 nm, grey), chlorophyll absorbance (680 nm, green), and Katushka fluorescence (645 nm, red) following addition of norflurazon at time zero. (**A**) pB10 transformant lacking Katushka. (**B**) AKL and (**C**) DKL transformants expressing Katushka. RFU—relative fluorescence units. Average of three replicates with standard errors shown.

Chlorophyll capture of the red photons emitted by Katushka is an essential step for enhancing the photosynthetic action spectrum. This was investigated by removing chlorophyll which would be predicted to increase Katushka fluorescence. The chlorophyll content of cells was reduced with 50 μM norflurazon [45]. Cells were grown mixotrophically in TAP medium containing acetate [34] under continuous cool white illumination. Chlorophyll absorbance (688 nm), Katushka fluorescence (645 nm), and cell density (750 nm) were measured for a period of 72 h following the addition of norflurazon (Figure 4).

Chlorophyll absorbance started to decrease after 24 h, dropping more rapidly from 32 to 44 h, and was not detectable after 72 h in all strains. At time zero, red 645 nm fluorescence was approximately six-fold higher from AKL and DKL transplastomic strains than the control pB10 strain (Figure 4A–C), consistent with incomplete masking of Katushka fluorescence by chlorophyll in these strains. Red fluorescence peaked at 40 h in AKL and DKL strains when chlorophyll absorbance had dropped by over 50%. Red fluorescence then dropped rapidly in concert with the sharp drop in chlorophyll absorbance. These results were consistent with the presence of Katushka in its active state, releasing red photons that were absorbed by chlorophyll. Prolonged photo-bleaching damages chloroplasts, and the associated changes, including protein degradation, most likely account for the drop in Katushka fluorescence observed after 40 h. The low red autofluorescence in the pB10 strain lacking Katushka closely followed the pattern of decline of chlorophyll absorbance (Figure 4A). OD_750_ nm readings were used to monitor changes in cell densities and showed comparable results for all three strains. Cell densities remained static for the first 40 h and then showed a relatively steady and moderate decrease in all three strains (Figure 4A–C).

### 3.4. CpKat Chloroplast Transformants Use Yellow Light for Photosynthesis

**Figure 5:**
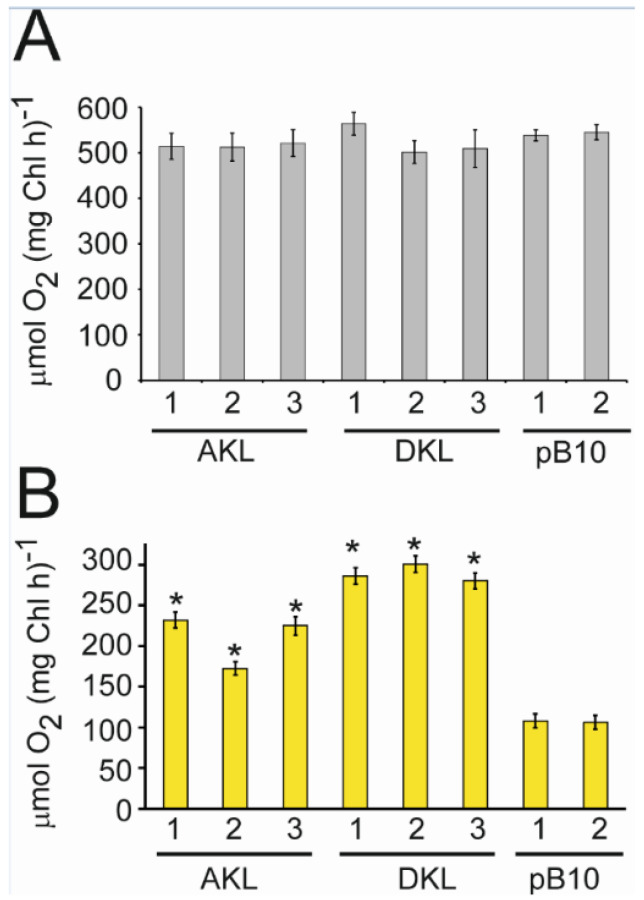
Photosynthetic oxygen evolution in AKL, DKL, and control pB10 (empty vector) transplastomic lines. Oxygen evolution following six hours of exposure of cells to 30 µmol m^−2^ s^−1^ of (**A**) cool white light, (**B**) yellow light. Average of three replicates with standard errors shown. Asterisks represent significant differences between the Katushka-expressing lines and the control pB10 line lacking the CpKAT gene (* *p* < 0.05).

Photosynthesis was monitored by oxygen evolution [47]. Equal amounts of AKL, DKL, and pB10 cells in minimal medium were placed in cool white light or yellow light (λ_max_ = 590) for six hours. Figure 5 shows the amount of dissolved oxygen found in the cultures. Similar dissolved oxygen concentrations were found in the AKL, DKL, and pB10 cultures exposed to cool white light (Figure 5A). Under yellow light, clear differences were found between the AKL and DKL cultures expressing Katushka and the negative pB10 control cultures lacking CpKat (Figure 5B). The results show that expression of Katushka in chloroplasts enables the utilisation of the energy in yellow photons to split water in photosystem II.

### 3.5. Yellow Light Promotes the Growth of Transplastomic Lines Expressing Katushka

**Figure 6:**
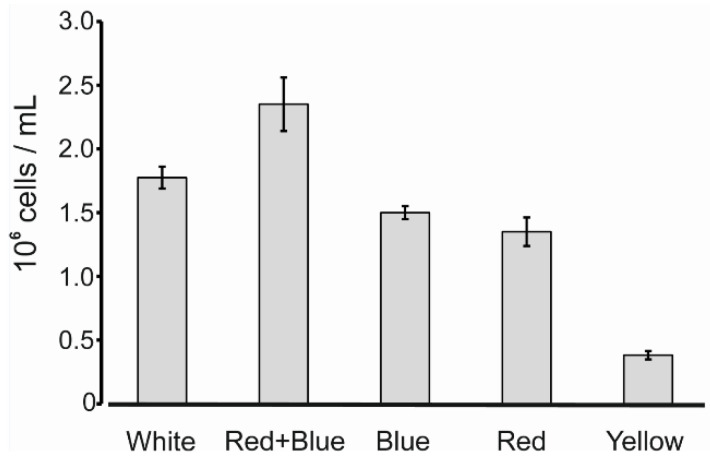
The influence of light conditions on the photosynthetic growth of *C. reinhardtii* CC-503 containing a wild-type chloroplast genome. Cell densities were measured two weeks after inoculating minimal media cultures with an equal number of cells and exposure to continuous light. White: 70 µmol m^−2^ s^−1^ of cool white fluorescent lamps. Red + Blue: 50 µmol m^−2^ s^−1^ of red light and 50 µmol µmol m^−2^ s^−1^ of blue light. Blue: 50 µmol m^−2^ s^−1^ of blue light. Red: 50 µmol m^−2^ s^−1^ of red light. Yellow: 30 µmol m^−2^ s^−1^ of yellow light. Average values from three replicate cultures with standard errors shown.

The influence of light conditions on growth of a control photosynthetic strain CC-503 lacking Katushka was determined (Figure 6). Under the conditions tested the CC-503 strain grew best in a mixture of red and blue light compared to red or blue light alone. These results showed that under the blue light conditions used, addition of red light boosted cell growth. Although differences in cell sizes and division rates have been reported for cultures grown in blue light versus red light [51,52] the final cell densities of CC-503 cultures grown in our blue and red light conditions were not significant (Figure 6). Growth of CC-503 in our standard cool white light conditions for propagating strains was reduced compared to combined blue and red light. The difference most probably reflects the lower energy provided by the white light conditions (70 μmol m^−2^ s^−1^) versus the combined blue and red light (100 μmol m^−2^ s^−1^). CC-503 grew relatively slowly in yellow light (30 μmol m^−2^ s^−1^).

**Figure 7:**
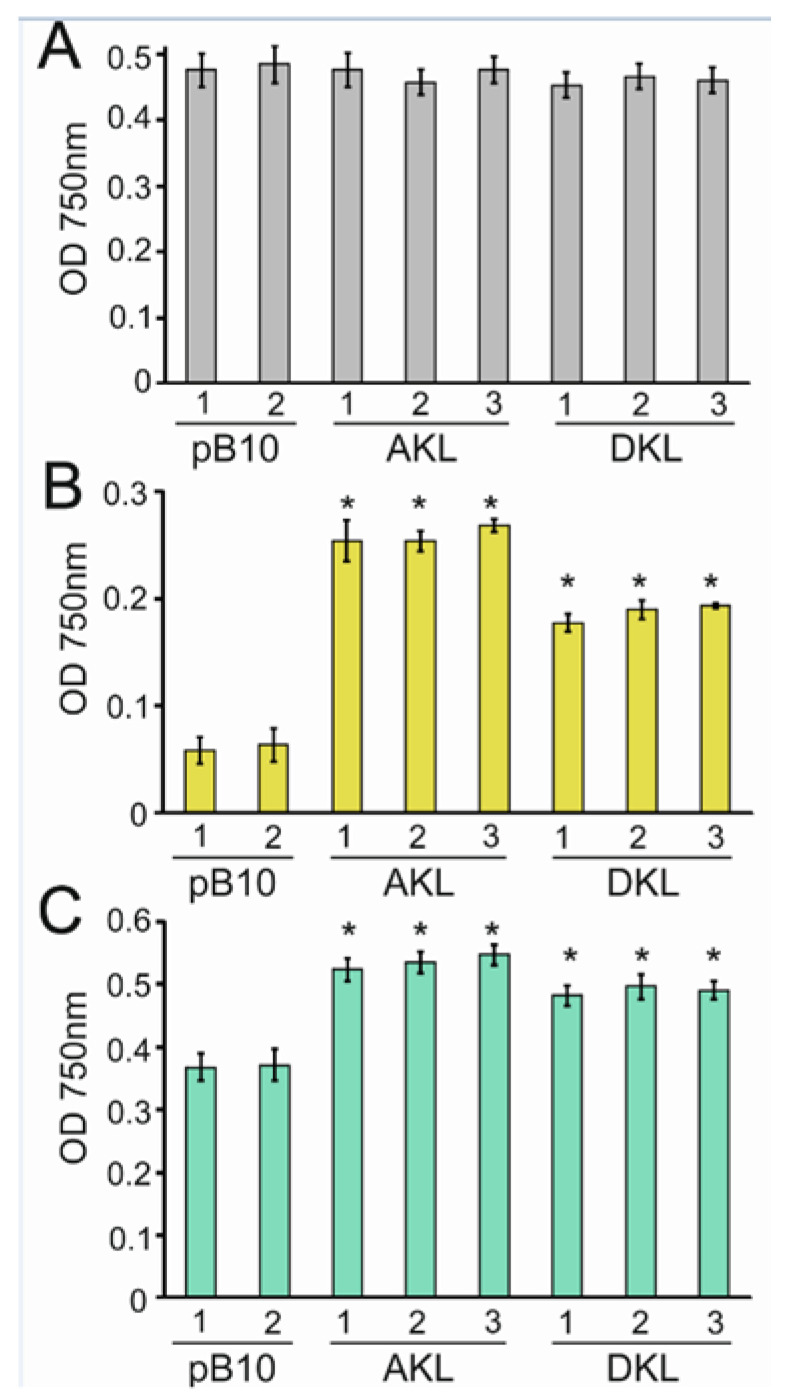
The influence of light conditions on the photosynthetic growth of transplastomic strains expressing Katushka (AKL and DKL) versus the control strain lacking Katushka (pB10). Cell densities were measured two weeks after inoculating minimal media cultures with an equal number of cells and exposure to continuous light. (**A**) 30 µmol m^−2^ s^−1^ of cool white light. (**B**) 30 µmol m^−2^ s^−1^ of yellow light. (**C**) 30 µmol m^−2^ s^−1^ blue light and 30 µmol m^−2^ s^−1^ yellow light. Average from three replicate cultures are presented with standard errors. Asterisks represent significant differences between the Katushka-expressing lines and the control pB10 line lacking the CpKAT gene (* *p* < 0.05).

Figure 7 shows growth of the AKL, DKL and control pB10 transplastomic strains in three different light regimes. All strains grew to comparable final densities in cool white light (Figure 7A), which contains blue and red photons. This shows that the presence of Katushka was not advantageous when red light was provided. The result also shows no detectable growth penalty or metabolic load associated with the expression of Katushka in chloroplasts. In yellow light (30 μmol m^−2^ s^−1^), the presence of Katushka had a clear positive impact on the growth of the AKL and DKL strains relative to the pB10 strain (Figure 7B). The final cell densities of AKL cultures were 4-fold, and DKL cultures 3-fold higher than the pB10 cultures. The densities of the pB10 cultures had dropped from the starting OD 750 nm values of 0.1. The growth of AKL and DKL cells can be explained by the conversion of yellow photons, which are poor drivers of photosynthesis, to red photons that are efficiently captured and used for photosynthesis. When yellow light (30 μmol m^−2^ s^−1^) was combined with blue light (30 μmol m^−2^ s^−1^), growth of all strains was increased relative to yellow light alone (Figure 7B,C) illustrating the higher quality of blue light compared to yellow light for photosynthetic growth. However, the AKL and DKL strains grew to higher densities than the pB10 strain in combined blue and yellow light (Figure 7C). This increased growth can be explained by the Katushka-mediated conversion of yellow to red photons in AKL and DKL strains. The combined results show that enhancing the photosynthetic action spectrum by using a device that can convert yellow photons to red photons improved photosynthetic growth of algal cells in the absence of red light when yellow light was provided.

## 4. Discussion

The fluorescent protein Katushka provided a simple light-capturing device to enhance the light spectrum for photosynthesis by converting yellow photons to red photons. This was exemplified by expressing Katushka in *C. reinhardtii* chloroplasts, which resulted in increased oxygen evolution and photosynthetic growth when cells were grown in yellow light. Moreover, chloroplast-localised Katushka increased the growth of algal cells grown in blue and yellow light. In white light, no differences in growth rate were observed between cells containing or lacking Katushka. In this situation, the abundance of red photons in white light would have masked those generated by Katushka fluorescence. These results strongly support Katushka-mediated conversion of yellow to red photons as the mechanism responsible for promoting cell growth when red light was absent. In effect, Katushka is acting as an accessory protein to capture yellow photons for photosynthesis. This synthetic biology approach involving redesigning algal cells to enhance the photosynthetic action spectrum complements other methods to boost photosynthesis, which include the use of submerged lighting [53] and fluorescent nanomaterials [54].

Katushka increases the number of recombinant fluorescent proteins expressed in *C. reinhardtii* [55]. Fluorescent proteins have been used mainly as reporters of gene expression and as protein tags for subcellular localisation [55,56,57]. As far as we are aware, this is the first use of a recombinant fluorescent protein to enhance the photosynthetic action spectrum. We used a coding sequence comprising frequently used chloroplast codons, a strategy that had previously been used to enhance accumulation of the green fluorescent protein in *C. reinhardtii* chloroplasts by 80-fold [57]. The CpKat expression cassettes were integrated close to the *atpB* gene in the intergenic region between the *rrnS* and *atpB* genes, which has been previously used to integrate foreign genes into chloroplast DNA [58]. We used the promoter and 5′ UTR regions from the chloroplast *atpA* and *psbD* genes, together with the 3′ region of the chloroplast *rbcL* gene, to express Katushka. These 5′ regulatory regions from the highly expressed *atpA* and *psbD* genes were chosen because they have been shown to be effective in driving the expression of recombinant proteins in chloroplasts [42,58,59]. Both sets of regulatory elements led to the accumulation of Katushka in chloroplasts and improved growth of transplastomic lines when yellow light was present and red light was absent. Increasing the amount and timing of Katushka expression, to fluctuate in response to the availability of yellow and red light, provides an approach to further improve growth. This will require developing new synthetic regulatory elements, sensitive to red and yellow light, to replace the native chloroplast regulatory elements used in this work.

Red photons emitted by Katushka were absorbed by chlorophyll. This was shown by the increase in red fluorescence emitted from cultures following removal of chlorophyll using the bleaching herbicide norflurazon. Transfer of fluorescent energy from Katushka to chlorophyll was likely to involve radiative energy transfer, rather than the more efficient fluorescence resonance energy transfer (FRET) which requires very small intermolecular distances [60]. Inefficient energy transfer to chlorophyll resulted in escape of Katushka-emitted red photons from cells, which was readily detected in transplastomic cell cultures expressing Katushka. Reducing the distance travelled by red photons would require targeting and accommodating Katushka in the crowded thylakoid membranes in chloroplasts that contain the chlorophyll light-harvesting complexes [61]. The potential of fluorescent proteins as light-capturing devices for photosynthesis will benefit from the identification of new brighter and higher quantum yield fluorescent proteins with favourable excitation and emission wavelengths [29]. Here, we explored the use of yellow photons to drive photosynthesis but the approach is applicable to other underutilised wavelengths in photosynthetic active radiation, which varies between taxa [19]. Combining fluorescent proteins with different excitation and emission wavelengths will increase the wavelength range of photons that can be captured.

## 5. Conclusions

The results show that a simple light-capturing device, comprising the Katushka fluorescent protein expressed in chloroplasts, improves photosynthetic growth of *C. reinhardtii* in yellow light alone, or in a mixture of blue and yellow light. The device works by converting yellow photons to red photons that are absorbed by the light-harvesting chlorophyll complexes in thylakoids to drive photosynthesis. This demonstrates the potential of enhancing the photosynthetic action spectrum to improve algal biomass accumulation under conditions where red light limits growth. This proof-of-concept study based on fluorescence of a single protein supports the utility of developing synthetic light-capture systems for photosynthesis [16,18,19,20]. Improving light-capture efficiencies at multiple wavelengths that are tunable to the environment is a desirable feature that will require more complex light-capture systems and advanced genetic engineering. This is likely to require multi-subunit complexes whose abundance is regulated by the quality and intensity of incident light.

## Data Availability

All data was presented within the paper and supplementary material.

## Supplementary Materials

The following are available online at https://www.mdpi.com/article/10.3390/microorganisms10091770/s1, Figure S1: DNA sequences of (**A**) AKL and (**B**) DKL chloroplast expression cassettes. The 5′ *atpA* [42], 5′ *psbD,* and 3′ *rbcL* regulatory regions and CpKat coding regions are indicated. Amino acid sequence of CpKat and restriction enzymes sites (double underlined) are shown. Table S1. Sequences of oligonucleotides used for assembling CpKat and PCR analyses.
