# Supplementary material for: A Chloroplast-Localised Fluorescent Protein Enhances the Photosynthetic Action Spectrum in Green Algae
Source: Microorganisms. 2022 Sep 1;10(9):1770. doi: 10.3390/microorganisms10091770 (PMC9504678; doi:10.3390/microorganisms10091770)
Supplement: Supplementary file 1 [file microorganisms-10-01770-s001.zip › microorganisms-1859851-supplementary.pdf]

A

AKL

Eco RI

atpA 5’ promoter and 5’ UTR

-2 GAATTCGATATCAAGCTTATCGATGACTTTATTAGAGGCAGTGTTTATATACCTAAACGTCAAAAGTCATTTTTATAACTGGTCTCAAAATACCTATAAAC
100 CCATTGTTCTTCTCTTTTAGCTCTAAGAACAATCAATTTATAAATATATTTATTATTATGCTATAATATAAATACTATATAAATACATTTACCTTTTTA
199 TAAATACATTTACCTTTTTTTTAATTTGCATGATTTTAATGCTTATGCTATCTTTTTTATTTAGTCCATAAAACCTTTAAAGGACCTTTTCTTATGGGA
298 TATTTATATTTTCCTAACAAAGCAATCGGCGTCATAAACTTTAGTTGCTTACGACGCCTGTGGACGTCCCCCCCTTCCCCTTACGGGCAAGTAAACTTA
397 GGGATTTTAATGCAATAAATAAATTTGTCCTCTTCGGGCAAATGAATTTTAGTATTTAAATATGACAAGGGTGAACCATTACTTTTGTTAACAAGTGAT
496 CTTACCACTCACTATTTTTGTTGAATTTTAAACTTATTTAAAATTCTCGAGAAAGATTTTAAAAATAAACTTTTTTAATCTTTTATTTATTTTTTCTTT

Nco I

M G S V L
595 TTTATGGCAATGCGTACTCCAGAAGAACTTAGTAATCTTATTAAAGATTTAATTGAACAATACACTCCAGAAGTGAAAATGTCCATGGGTTCAGTTTTA
I T E N M H M K L Y M E G T V N D H H F K C T S E G E G K P Y E G
694 ATTACAGAAAATATGCATATGAAATTATATATGGAAGGTACAGTTAATGATCATCATTTTAAATGTACATCAGAAGGTGAAGGTAAACCATATGAAGGT
T Q T M K I K V V E G G P L P F A F D I L A T S F M Y G S K T F I
793 ACACAAACAATGAAAATTAAAGTTGTTGAAGGTGGTCCATTACCATTTGCTTTTGATATTTTAGCTACATCATTTATGTATGGTTCAAAAACATTTATT
N H T Q G I P D F F K Q S F P E G F T W E R I T T Y E D G G V L T
892 AATCATACACAAGGTATTCCAGATTTTTTTAAACAATCATTTCCAGAAGGTTTTACATGGGAACGTATTACAACATATGAAGATGGTGGTGTTTTAACA
A T Q D T S L Q N G C L I Y N V K I N G V N F P S N G P V M Q K K
991 GCTACACAAGATACATCATTACAAAATGGTTGTTTAATTTATAATGTTAAAATTAATGGTGTTAATTTTCCATCAAATGGTCCAGTTATGCAAAAAAAA
T L G W E A S T E M L Y P A D S G L R G H S Q M A L K L V G G G Y
1090 ACATTAGGTTGGGAAGCTTCAACAGAAATGTTATATCCAGCTGATTCAGGTTTACGTGGTCATTCACAAATGGCTTTAAAATTAGTTGGTGGTGGTTAT
L H C S L K T T Y R S K K P A K N L K M P G F Y F V D R R L E R I
1189 TTACATTGTTCATTAAAAACAACATATCGTTCAAAAAAACCAGCTAAAAATTTAAAAATGCCAGGTTTTTATTTTGTTGATCGTCGTTTAGAACGTATT

PstI

K E A D K E T Y V E Q H E M A V A R Y C D L P S K L G H S * BamHI
1288 AAAGAAGCTGATAAAGAAACATATGTTGAACAACATGAAATGGCTGTTGCTCGTTATTGTGATTTACCATCAAAATTAGGTCATTCATAACTGCAGGAT

BamHI

rbcL 3’ UTR region

1387 CCTCTAGATTTAATTTTTATTTTTCATGATGTTTATGTGAATAGCATAAACATCGTTTTTATTTTTTATGGTGTTTAGGTTAAATACCTAAACATCATT
1486 TTACATTTTTAAAATTAAGTTCTAAAGTTATCTTTTGTTTAAATTTGCCTGTGCTTTATAAATTACGATGTGCCAGAAAAATAAAATCTTAGCTTTTTA

NotI

1585 TTATAGAATTTATCTTTATGTATTATATTTTATAAGTAATAAAAGAAATAGTAACATACTAAAGCGGATGTAACTCAATCGCGGCCGC

B DKL

```
    Eco RI/HincII blunt                 psbD 5' promoter and 5' UTR
  -2 GAATTGACGTATTAGTTGTAAACTTGACTAACATTTTAAATTTTAAATTTTTTCCTAATTATATATTTTACTTGCAAAATTTATAAAAATTTTATGCATTT
 100 TTATATCATAATAATAAAACCTTTATTCATGGTTTATAATACAATAATTGTGATGACTATGCACAAAGCAGTTCTAGTCCCATATATATAACTATATAT
 199 AACCCGTTTAAAGATTTATTTAAAAATATGTGTGTAAAAAATGCTTATTTTTAATTTTATTTTATATAAGTTATAATATTAAATACACAATGATTAAAA

                                                                                                       Nco I
                                                                                                          M  G  S  V  L  I  T  E
 298  TTAAATAATAATAAATTTAACGTAACGATGAGTTGTTTTTTTATTTTGGAGATACACGCAATGACAATTGCGTCCATGGGTTCAGTTTTAATTACAGAA
      N  M  H  M  K  L  Y  M  E  G  T  V  N  D  H  H  F  K  C  T  S  E  G  E  G  K  P  Y  E  G  T  Q  T
 397  AATATGCATATGAAATTATATATGGAAGGTACAGTTAATGATCATCATTTTAAATGTACATCAGAAGGTGAAGGTAAACCATATGAAGGTACACAAACA
      M  K  I  K  V  V  E  G  G  P  L  P  F  A  F  D  I  L  A  T  S  F  M  Y  G  S  K  T  F  I  N  H  T
 496  ATGAAAATTAAAGTTGTTGAAGGTGGTCCATTACCATTTGCTTTTGATATTTTAGCTACATCATTTATGTATGGTTCAAAAACATTTATTAATCATACA
      Q  G  I  P  D  F  F  K  Q  S  F  P  E  G  F  T  W  E  R  I  T  T  Y  E  D  G  G  V  L  T  A  T  Q
 595  CAAGGTATTCCAGATTTTTTTAAACAATCATTTCCAGAAGGTTTTACATGGGAACGTATTACAACATATGAAGATGGTGGTGTTTTAACAGCTACACAA
      D  T  S  L  Q  N  G  C  L  I  Y  N  V  K  I  N  G  V  N  F  P  S  N  G  P  V  M  Q  K  K  T  L  G
 694  GATACATCATTACAAAATGGTTGTTTAATTTATAATGTTAAAATTAATGGTGTTAATTTTCCATCAAATGGTCCAGTTATGCAAAAAAAAACATTAGGT
      W  E  A  S  T  E  M  L  Y  P  A  D  S  G  L  R  G  H  S  Q  M  A  L  K  L  V  G  G  G  Y  L  H  C
 793  TGGGAAGCTTCAACAGAAATGTTATATCCAGCTGATTCAGGTTTACGTGGTCATTCACAAATGGCTTTAAAATTAGTTGGTGGTGGTTATTTACATTGT
      S  L  K  T  T  Y  R  S  K  K  P  A  K  N  L  K  M  P  G  F  Y  F  V  D  R  R  L  E  R  I  K  E  A
 892  TCATTAAAAACAACATATCGTTCAAAAAAACCAGCTAAAAATTTAAAAATGCCAGGTTTTTATTTTGTTGATCGTCGTTTAGAACGTATTAAAGAAGCT

                                                                                                                PstI
      D  K  E  T  Y  V  E  Q  H  E  M  A  V  A  R  Y  C  D  L  P  S  K  L  G  H  S                  BamHI.
 991 GATAAAGAAACATATGTTGAACAACATGAAATGGCTGTTGCTCGTTATTGTGATTTACCATCAAAATTAGGTCATTCATAACTGCAGGATCCTCTAGAT

                 rbcL 3' UTR region
1090 TTAATTTTTATTTTTCATGATGTTTATGTGAATAGCATAAACATCGTTTTTATTTTTTATGGTGTTTAGGTTAAATACCTAAACATCATTTTACATTTT
1189 TAAAATTAAGTTCTAAAGTTATCTTTTGTTTAAATTTGCCTGTGCTTTATAAATTACGATGTGCCAGAAAAATAAAATCTTAGCTTTTTATTATAGAAT

                                                                                                       NotI
1288 TTATCTTTATGTATTATATTTTATAAGTAATAAAAGAAATAGTAACATACTAAAGCGGATGTAACTCAATCGCGGCCGC
```

Supplementary Figure S1. DNA sequences of (**A**) AKL and (**B**) DKL chloroplast expression cassettes. The 5' *atpA* [42], 5' *psbD* and 3' *rbcL* regulatory regions, and CpKat coding regions are indicated. Amino acid sequence of CpKat and restriction enzymes sites (double underlined) are shown

Supplementary Table S1: Sequences of oligonucleotides used

Oligo	Sequence 5' → 3'
782	GGGATCCTTGCCATGGGTTCAGTTTTAATTACAG
783	ATGGGTTCAGTTTTAATTACAGAAAATATGCATATGAAATT
784	ATTAACTGTACCTTCCATATATAATTTCATATGC
785	TGGAAGGTACAGTTAATGATCATCATTTTAAATGT
786	ATGGTTTACCTTCACCTTCTGATGTACATTTAAAA
787	AGGTGAAGGTAAACCATATGAAGGTACACAAACAA
788	CCACCTTCAACAACTTTAATTTTCATTGTTTGTGT
789	AAAGTTGTTGAAGGTGGTCCATTACCATTTGCTTT
790	CATAAATGATGTAGCTAAAATATCAAAAGCAAATG
791	TAGCTACATCATTTATGTATGGTTCAAAAACATTT
792	CTGGAATACCTTGTGTATGATTAATAAATGTTTTT
793	TACACAAGGTATTCCAGATTTTTTTAAACAATCAT
794	CGTTCCCATGTAAAACCTTCTGGAAATGATTGTTT
795	GGTTTTACATGGGAACGTATTACAACATATGAAGA
796	TTGTGTAGCTGTTAAAACACCACCATCTTCATATG
797	TTTTAACAGCTACACAAGATACATCATTACAAAAT
798	TTTTAACATTATAAATTAAACAACCATTTTGTAAT
799	AATTTATAATGTTAAAATTAATGGTGTTAATTTTC
800	TTTTGCATAACTGGACCATTTGATGGAAAATTAAC
801	GGTCCAGTTATGCAAAAAAAAACATTAGGTTGGGA
802	TGGATATAACATTTCTGTTGAAGCTTCCCAACCTA
804	CAGAAATGTTATATCCAGCTGATTCAGGTTTACGT
805	ATTTTAAAGCCATTTGTGAATGACCACGTAAACCT
806	ACAAATGGCTTTAAAATTAGTTGGTGGTGGTTATT
807	TATGTTGTTTTTAATGAACAATGTAAATAACCACC

808	TCATTAAAAACAACATATCGTTCAAAAAAACCAGC
809	AAAACCTGGCATTTTTAAATTTTTAGCTGGTTTTT
810	TAAAAATGCCAGGTTTTTATTTTGTTGATCGTCGT
811	TATCAGCTTCTTTAATACGTTCTAAACGACGATCA
812	TATTAAAGAAGCTGATAAAGAAACATATGTTGAAC
813	TAACGAGCAACAGCCATTTCATGTTGTTCAACATA
814	ATGGCTGTTGCTCGTTATTGTGATTTACCATCAAA
815	TTGCATTCCTGCAGTTATGAATGACCTAATTTTGATGG
862	TTCTAGAGGGCCCTGAATTCAAAGTGGTACAATAAATAAATTG
863	GGGTACCAACACAACAATTAACTAAAGTG
864	CCTGCAGGATCCTCTAGATTTAATTTTTATTTTTCATGATG
865	GGCGGCCGCGATTGAGTTACATCCGCTTTAG
1036	GGAGCTCCGTATTACTATTCGTATTAGG
1038	GGGGAAGGAGGAGGTTCTTATTTCAAAT
1123	TGCGGCCGCTGGGGGT
1124	CTAGACCCCCAGCGGCCGCAAGCT
1125	CCCCCACCCCC
1126	GATCGGGGGTGGGGGGC
1420	GTTGGAGACCTTCAAGCCGT
5199a	GGGTCGACGTATTAGTTGTAAACTTGAC
5199b	CCCCATGGACGCAATTGTCATTGCGTG
